# Impact of coronavirus disease 2019 on urgent referrals to secondary care otolaryngology: a prospective case series

**DOI:** 10.1017/S0022215120002091

**Published:** 2020-09-28

**Authors:** M S Osborne, E Bentley, A Farrow, J Chan, J Murphy

**Affiliations:** 1 Department of ENT, New Cross Hospital, Wolverhampton, UK; 2 Department of ENT, Princess Royal Hospital, Telford, UK

**Keywords:** Coronavirus, Referral, Primary Care, Emergency Care

## Abstract

**Objective:**

As the novel coronavirus disease 2019 changed patient presentation, this study aimed to prospectively identify these changes in a single ENT centre.

**Design:**

A seven-week prospective case series was conducted of urgently referred patients from primary care and accident and emergency department.

**Results:**

There was a total of 133 referrals. Referral rates fell by 93 per cent over seven weeks, from a mean of 5.4 to 0.4 per day. Reductions were seen in referrals from both primary care (89 per cent) and the accident and emergency department (93 per cent). Presentations of otitis externa and epistaxis fell by 83 per cent, and presentations of glandular fever, tonsillitis and peritonsillar abscess fell by 67 per cent.

**Conclusion:**

Coronavirus disease 2019 has greatly reduced the number of referrals into secondary care ENT. The cause for this reduction is likely to be due to patients’ increased perceived risk of the virus presence in a medical setting. The impact of this reduction is yet to be ascertained, but will likely result in a substantial increase in emergency pressures once the lockdown is lifted and the general public's perception of the coronavirus disease 2019 risk reduces.

## Introduction

In December 2019, a pneumonia outbreak was reported in Wuhan, Hubei province, China, and was subsequently named 2019 novel coronavirus (‘2019-nCoV’) or coronavirus disease 2019 (Covid-2019) by the World Health Organization (WHO). This disease spread globally, affecting over 3.2 million people and resulting in more than 233 000 deaths as of 1st May 2020.^1^ The virus itself closely resembles severe acute respiratory syndrome and Middle East respiratory syndrome viruses, and has been named severe acute respiratory syndrome coronavirus-2 (SARS-CoV-2).^2^ On 20th January, the WHO declared a public health emergency, and on 29th January 2020 the first two patients tested positive for coronavirus in the UK. On 28th February 2020, the first case of internal transmission within the UK was reported.

During this time, the ENT department of New Cross Hospital, Wolverhampton, West Midlands, was undertaking a study into referrals to our secondary care centre and this provided an excellent opportunity to review the direct impact of Covid-19. The current study aimed to review the effect of Covid-19 on referrals to a single secondary centre over a seven-week period.

## Materials and methods

A prospective case series review was undertaken of all referrals from primary care and local accident and emergency (A&E) department to the on-call ENT junior doctor. Data were collected over a seven-week period from 10th February to 29th March 2020, at which point junior doctors were redeployed to assist in other areas of the hospital. Data were obtained for all urgent referrals during this period. The receiving clinicians were always ENT doctors, as no specialty cross-cover occurs out of hours. Any referrals directly to the on-call ENT registrar were excluded from this study. Patient demographics, referral time and referrers’ diagnoses were recorded.

A standardised proforma was initiated for each patient upon referral, and this form was subsequently completed at the time of review by the ENT junior doctor. The form was completed when the patient attended either the A&E department or the rapid access clinic. An assessment of the effects and patterns on referral rates of seven key events that occurred during this study period, was undertaken.

## Results

Over the seven-week study period, the ENT department received 133 referrals directly from primary care or A&E. Thirty-six referrals (27 per cent) were received from primary care, of which 42 per cent (*n* = 15) were seen on the same day. Ninety-seven patients (73 per cent) were referred from the A&E department and 82 per cent (*n* = 80) of these were seen directly. Fourteen per cent (*n* = 14) received an appointment for the rapid access clinic and 3 per cent (*n* = 3) received telephone clinical advice only. The most common presentations were otitis externa, otitis media, epistaxis, peri-tonsillar abscess and tonsillitis (Table 1).

**Table 1.** Frequency of diagnoses as reported by referring clinician

Diagnosis	Cases (*n*)
Ear	
– Otitis externa	17
– Otitis media	16
– Mastoiditis	5
– Aural foreign body	4
– Pinna laceration	4
– Pinna haematoma	2
– TM perforation	2
– MOE	2
– Impacted wax	1
– Bell's palsy	1
Nose	
– Epistaxis	15
– Nasal foreign body	2
– Nasal laceration	3
– Septal haematoma	1
– Acute sinusitis	1
– Peri-orbital cellulitis	1
– Nasal fracture	1
Throat or head & neck	
– Quinsy	22
– Tonsillitis	13
– Foreign body throat	5
– Airway compromise	5
– Neck abscess	3
– Food bolus	2
– SCC	2
– Epiglottitis or supraglottitis	1
– Post-tonsillectomy bleed	1
– Parotitis	1

TM = tympanic membrane; MOE = malignant otitis externa; SCC = squamous cell carcinoma

Over the seven-week study period, daily referral rates fell by 93 per cent, from a peak mean of 5.4 to 0.4 per day. Weekly referral rates declined by 92 per cent, from a peak of 38 to 3 per week following the implementation of UK lockdown (Table 2).

**Table 2.** Numbers of patients referred to ENT department during data collection period

Referrals by week (date)	Monday	Tuesday	Wednesday	Thursday	Friday	Saturday	Sunday	Total referrals	Daily mean	EDSS total A&E attendance in England
Week 1 (10/02/20)	10	6	5	1	2	7	3	34	4.9	173 447
Week 2 (17/02/20)	9	10	5	0	3	4	7	38	5.4	152 693
Week 3 (24/02/20)	1	4	2	2	0	0	3	12	1.7	177 370
Week 4 (02/03/20)	1	2	1	5	1	2	2	14	2.0	174 428
Week 5 (09/03/20)	4	6	2	0	1	1	0	14	2.0	153 105
Week 6 (16/03/20)	1	3	3	3	2	5	1	18	2.6	120 356
Week 7 (23/03/20)	0	0	0	1	2	0	0	3	0.4	89 584

Data represent numbers of patients. EDSS = Emergency Department Syndromic Surveillance System; A&E = accident and emergency department

**Fig. 1.**
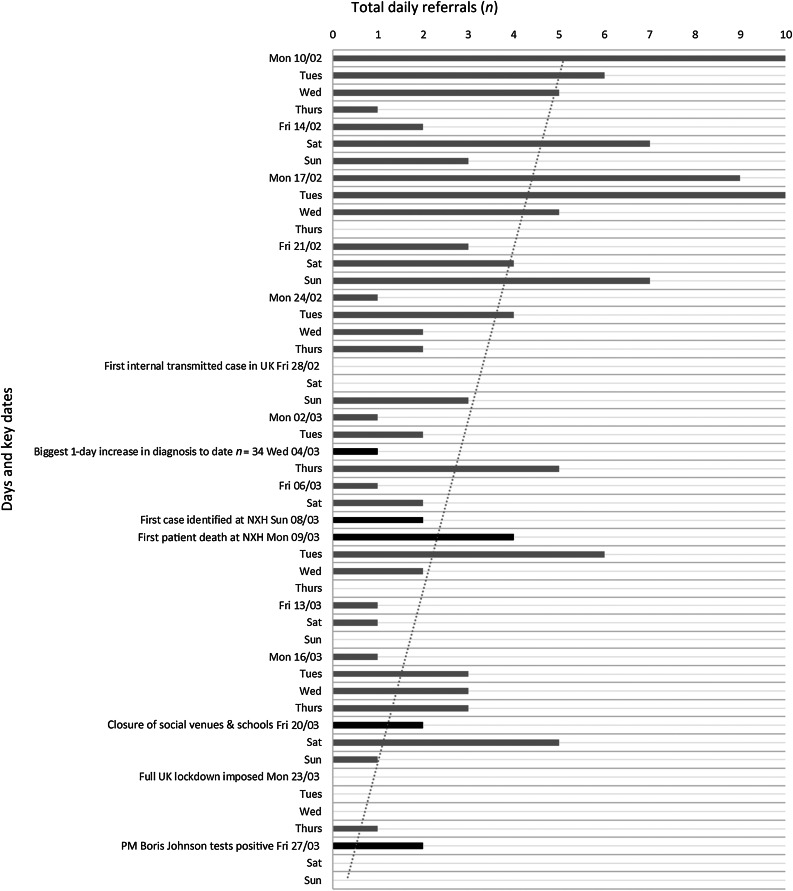
Numbers of daily referrals over the study period. Key dates are shown in bold. NXH = New Cross Hospital, Wolverhampton; PM = Prime Minister

Figure 1 demonstrates the daily referrals received over the study period. During this time, seven key events took place. First, on 28th February 2020, the first British victim died of Covid-19 on board the Diamond Princess cruise ship. UK authorities confirmed the first case of the illness due to local transmission. Second, on 4th March 2020, cases of Covid-19 surged in the UK, the biggest 1-day increase so far (34 cases), bringing the total number of UK cases to 87. Third, on 8th March 2020, the first case was identified in New Cross Hospital, Wolverhampton, UK. Fourth, on 9th March 2020, the first Covid-19 related death occurred at New Cross Hospital. Fifth, on 20th March 2020, the UK government ordered all pubs, restaurants, gyms and other social venues across the country to close. Sixth, on 23rd March 2020, lockdown was imposed. Britons were told to only go outside to buy food, exercise (once a day) or to go to work if they absolutely could not work from home. Seventh, on 27th March 2020, the UK Prime Minister Boris Johnson and Health Secretary Matt Hancock tested positive for the coronavirus.

Each of these events was widely reported in the media. In the two weeks prior to the first reported locally transmitted case of Covid-19, our department received a mean of 36 referrals per week (34 and 38 respectively). This fell by 60 per cent to a mean of 14.5 referrals per week for the subsequent four weeks. Following the imposition of the lockdown on the 23rd March, referrals reduced by a further 79 per cent, to three patients, in the subsequent 7 days. According to data released by the Emergency Department Syndromic Surveillance System over this same period, A&E attendance within England had fallen by 25.6 per cent, to 89 584, from 120 356 the previous week.^3,4^ Table 2 demonstrates the relationship between the total daily and weekly referral rates and the Emergency Department Syndromic Surveillance System data.

**Fig. 2.**
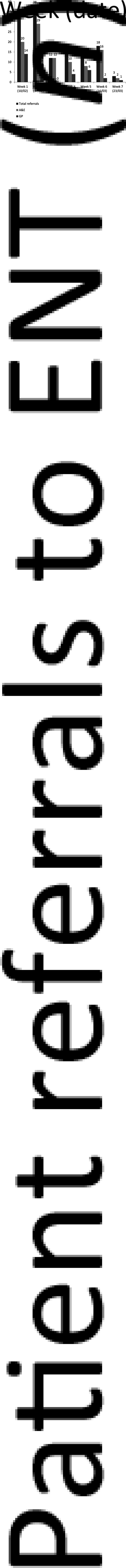
Numbers of weekly referrals and referral source (primary care vs accident and emergency department) over the study period. A&E = accident and emergency; GP = general practitioner

The A&E referrals accounted for 73 per cent (*n* = 97) and primary care for 27 per cent (*n* = 36) of all referrals over the total study period. Figure 2 shows how these referral rates for each of these two sources fluctuated throughout the study period. The most significant change was the reduction that occurred in week seven of the study following the initiation of lockdown. Referrals from primary care dropped 89 per cent, from 14 to 1 patient per week, and referrals from A&E fell by 93 per cent, from 29 to 2 patients per week.

## Discussion

Patients presenting with an ENT-related problem are commonly encountered in a primary care setting, and can comprise up to approximately one-quarter of adult consultations and 50 per cent of paediatric consultations.^5^ In A&E departments, ENT presentations account for 12.5 per cent of first recorded diagnoses.^6^ The significant reduction seen in referrals in this study cannot be solely accounted for by a reduction in the prevalence of disease or its sequelae, and is more likely to represent the psychosocial impact of coronavirus.

A significant change in patients’ behaviour will have been brought about by the daily government briefings advising the public to ‘protect the National Health Service’. This impact is compounded by social and mainstream media, and its depiction of the health services being stretched by the burden of the disease, both within the UK and abroad. The social consciousness in the UK may provide a protective response to the National Health Service, resulting in patients staying away to the detriment of themselves, as they perceive the needs of others as greater than their own. Patients may also be too anxious to attend a hospital site where they might contract Covid-19 and suffer its harmful effects.

A reduction in physical activities may decrease the occurrence of traumatic injuries to the nose or face. In addition, a reduction in social interaction, associated with self-isolation, will reduce the spread of all communicable diseases. This may account for the decrease in referrals for conditions such as: otitis externa (resulting from no swimming), which fell by 83 per cent; Glandular fever, tonsillitis and peritonsillar abscess, which decreased by 83 per cent, from a weekly mean of six patients to less than one patient. This is likely to be a result of government recommendations advising against touching the face, leading to less nasal mucosal irritation that may precipitate bleeding. However, these types of behaviour changes cannot explain the subjective reduction in the presentation of cancer patients to the ENT department.

As time passes and the lockdown is lifted, the implications of these delayed presentations will become more apparent. It is possible that referrals rates for both primary care and A&E will rebound, with patients presenting at a later stage of their disease when they do attend.

- Coronavirus disease 2019 reduced accident and emergency department attendance by 25 per cent in the first week following lockdown
- Referrals to the ENT department at New Cross Hospital, Wolverhampton, dropped by 93 per cent within the same period
- Otitis externa referrals fell by 83 per cent
- Glandular fever, tonsillitis and peritonsillar abscess referrals fell by 67 per cent

The significant difference between the national reduction in A&E attendance (26 per cent) and the drop in local referrals to ENT (93 per cent) is likely to represent regional variation at the time our data were collected. Hotspots across the UK affect regional presentations, with Wolverhampton having one of the highest rates of infection at the time of this study. This discrepancy may also be due to the excellent treatment given by the A&E department, preventing referrals to ENT at this time, possibly reflecting a reduction in time pressure within the A&E department related to the decrease in attendance.

## Conclusion

Overall, referral rates to the ENT department at New Cross Hospital, Wolverhampton, dropped by 92 per cent following the onset of the coronavirus pandemic. This reduction was seen in both primary care (89 per cent) and A&E (93 per cent) settings. The decrease in referrals is likely due to patients’ reluctance to attend for review and partly due to a reduction in the prevalence of some ENT-related conditions. The impact of these delayed presentations is yet to be ascertained, but a rebound in emergency pressures is possible once the lockdown has lifted and the general public's perception of Covid-19 risk reduces.

## References

[1] Johns Hopkins University & Medicine. Coronavirus Resource Center. In: https://coronavirus.jhu.edu/ [4 May 2020]

[2] Coronaviridae Study Group of the International Committee on Taxonomy of Viruses. The species Severe acute respiratory syndrome-related coronavirus: classifying 2019-nCoV and naming it SARS-CoV-2. Nat Microbiol 2020;5:536–44 32123347 10.1038/s41564-020-0695-z PMC7095448

[3] Public Health England. Emergency Department Syndromic Surveillance System: England. Year 2020, week 13. 2 April 2002. In: https://assets.publishing.service.gov.uk/government/uploads/system/uploads/attachment_data/file/877600/EDSSSBulletin2020wk13.pdf.pdf [4 May 2020]

[4] Public Health England. Emergency Department Syndromic Surveillance System: England. Year 2020, week 12. 26 March. In: https://assets.publishing.service.gov.uk/government/uploads/system/uploads/attachment_data/file/875604/EDSSSBulletin2020wk12__1_.pdf [4 May 2020]

[5] Griffiths E. Incidence of ENT problems in general practice. J R Soc Med 1979;72:740–2 552431 10.1177/014107687907201008 PMC1437201

[6] UK Parliament House of Commons Library. Accident and Emergency Statistics: Demands, Performance and Pressure 2017. In: https://commonslibrary.parliament.uk/research-briefings/sn06964/ [4 May 2020]

